# After Buffalo, Where?

**Published:** 1896-09

**Authors:** 


### AFTER BUFFALO—WHERE?

SURELY it was good to have been there; and after such a reception, such an intellectual feast, such a glorious reunion, so representative a gathering, so enjoyable a time, the exhibition of the utmost harmony and true professional spirit, we say after Buffalo—where? Not because of fear, but of deep concern; for joys and privileges, such as we have tasted and feasted of at the "Queen City of the Lakes" are yearned for at all times, and only a few of our great centres of population can afford such a measure of good things. From entrance reception to the small hours of the night, when we arose from the festive banquet board, it was a season of joy never to be forgotten by any of those present, and all honor to Buffalo and the Empire State; all praise to her worthy representatives of our profession, and may peace, harmony, and prosperity follow in the pathway of all who have so well conserved the interests of our national organization and added to it an era of greater strength and power than she has ever enjoyed. This is Buffalo's response to her appeal to come and taste of the joys of her beautiful and home-like city, and we raise our voice with one accord in sincere appreciation of the bounteous hospitality so freely accorded us. Let us not be unmindful of the great debt she has laid upon the shoulders of all future convention cities, and, with this uppermost in our minds, we will then measure fairly the ability and opportunities of other sections of our country where it will be necessary for us to go in the upbuilding of our profession and our Association, and be as generous in our exactions from other centres as Buffalo has been bounteous in her giving to us at this time. With Columbus, Cleveland, Detroit, Kansas City, and Nashville seeking our favor for 1897, we repeat: After Buffalo—where?

